# Stimulation through CD40 and TLR-4 Is an Effective Host Directed Therapy against *Mycobacterium tuberculosis*

**DOI:** 10.3389/fimmu.2016.00386

**Published:** 2016-09-27

**Authors:** Nargis Khan, Susanta Pahari, Aurobind Vidyarthi, Mohammad Aqdas, Javed N. Agrewala

**Affiliations:** ^1^CSIR-Institute of Microbial Technology, Chandigarh, India

**Keywords:** CD40, TLR-4, immunomodulation, innate immunity, dendritic cells, T cell response, autophagy

## Abstract

Tuberculosis (TB) is the leading cause of morbidity and mortality among all infectious diseases. Failure of *Bacillus Calmette Guerin* as a vaccine and serious side-effects and toxicity due to long-term TB drug regime are the major hurdles associated with TB control. The problem is further compounded by the emergence of drug-resistance strains of *Mycobacterium tuberculosis* (*Mtb*). Consequently, it demands a serious attempt to explore safer and superior treatment approaches. Recently, an improved understanding of host–pathogen interaction has opened up new avenues for immunotherapy for treating TB. Although, dendritic cells (DCs) show a profound role in generating immunity against *Mtb*, their immunotherapeutic potential needs to be precisely investigated in controlling TB. Here, we have devised an approach of bolstering DCs efficacy against *Mtb* by delivering signals through CD40 and TLR-4 molecules. We found that DCs triggered through CD40 and TLR-4 showed increased secretion of IL-12, IL-6, and TNF-α. It also augmented autophagy. Interestingly, CD40 and TLR-4 stimulation along with the suboptimal dose of anti-TB drugs significantly fortified their efficacy to kill *Mtb*. Importantly, animals treated with the agonists of CD40 and TLR-4 boosted Th1 and Th17 immunity. Furthermore, it amplified the pool of memory CD4 T cells as well as CD8 T cells. Furthermore, substantial reduction in the bacterial burden in the lungs was observed. Notably, this adjunct therapy employing immunomodulators and chemotherapy can reinvigorate host immunity suppressed due to drugs and *Mtb*. Moreover, it would strengthen the potency of drugs in curing TB.

## Introduction

Tuberculosis (TB) is one of the world’s most devastating disease killing 2 million people annually (1–3). Even though TB drugs are the backbone of current treatment that successfully eliminate *Mycobacterium tuberculosis* (*Mtb)*; their efficacy is limited by a narrow therapeutic index and chronic toxicities. Furthermore, the situation has worsened due to emergence of drug-resistant strains of *Mtb*. Thus, it is considerably important to discover effective therapeutic stratagems to treat TB.

Since last two decades, the paradigm shift in the treatment of various diseases, preferably tumors and autoimmune diseases have evolved from chemotherapeutic agents to selective and mechanism-based approaches (4). More recently, an improved understanding of host–pathogen interaction has given rise to new treatment options, which includes immunomodulatory approaches.

Dendritic cells (DCs) are the most potent antigen-presenting cells (APCs) (5, 6). By virtue of their efficient antigen presentation and elicitation of T cell response, several vaccination approaches have been developed to explore potency of DCs in enhancing the host immunity, particularly in the case of tumors (7). Noteworthy, tumors suppress DCs function to establish their growth (8, 9). However, DCs potent anti-tumor activity has been explored by reinvigorating their function by delivering signals through their surface receptors (7, 10). Consequently, indicating that impairment in the function of DCs can be restored. In addition to tumors, *Mtb* can also empower and impair the function of DCs (11). *Mtb*-infected DCs exhibit several deficiencies, including limited migration to draining lymph nodes, reduced lifecycle, and inferior bactericidal activity; thereby fail to elicit and impart protective immunity against *Mtb* (12, 13).

Dendritic cells express an array of costimulatory molecules, such as CD40, CD80, and CD86. These molecules can influence the activation of T cells and DCs (14). Furthermore, CD40 signaling can control the replication of viruses (15, 16). However, CD40 triggering can enhance the T cell response in *Mtb*-challenged mice but fails to reduce the bacterial burden in their lungs (17). This suggests that CD40 signaling needs some additional stimulator to enhance its efficacy. Toll-like receptors (TLRs) are known to amplify CD40-mediated immune response of DCs (7, 18, 19). The central role played by CD40 in eliciting acquired immunity is mirrored by TLR-4 in promoting innate immunity (20). Unlike other TLRs, TLR-4 in conjunction with CD40 maintains the persistence of IL-12 (7) that is crucial for the induction of Th1 immunity against *Mtb*. In addition, TLR-4 is a potent inducer of autophagy, which is a classical mechanism to restrict the intracellular survival of *Mtb* (21). Furthermore, it has been demonstrated that macrophages infected with live *Mtb* shows upregulation of genes induced by MyD88-independent signaling pathway. Thereby, TLR-mediated responses to *Mtb* involve mostly MyD88-independent pathway (22). Among all the TLRs, only TLR-4 drives signal through both MyD88-dependent and -independent pathways (23). Therefore, combinatorial signaling through CD40 and TLR-4 may be a prudent step in bolstering the host immunity to limit *Mtb* growth.

Taking into consideration of the above-mentioned facts, we thought to explore CD40 and TLR-4 (C40.T4)-mediated signaling to boost host immunity. Furthermore, the concerted action of C40.T4 in association with anti-TB drugs to eliminate *Mtb* was also studied. In the present study, we demonstrate that triggering of *Mtb*-infected DCs through C40.T4 elicited T cell response, augmented the release of IL-6, IL-12, and TNF-α and reduced the intracellular bacterial burden. Interestingly, it was noticed that adjunct therapy employing CD40 and TLR-4 agonists (C40.T4) significantly enhanced *in vitro* and *in vivo* killing potency of anti-TB drugs. The mechanism of augmented killing of mycobacterium in DCs was elucidated through involvement of autophagy. Thus, this study may open up new avenues of adjunct therapy employing drugs and C40.T4 to successfully treat TB.

## Materials and Methods

### Animals

C57BL/6 mice, 6–8 weeks were procured from the Institute of Microbial Technology (IMTECH), Chandigarh, India.

### Ethics Statement

All experiments were approved by the Institutional Animal Ethics Committee of IMTECH and performed according to the National Regulatory Guideline issued by Committee for the Purpose of Supervision of Experiments on Animals (No. 55/1999/CPCSEA), Ministry of Environment and Forest, Government of India and Institutional Biosafety Committee.

### Antibodies and Reagents

All the recombinant cytokines, antibodies (Abs) and ELISA reagents were purchased from BD Biosciences (San Diego, CA, USA) and unless mentioned. TLR-4L (LPS EK ultrapure) was procured from Invivogen (San Diego, CA, USA). Abs for Western blotting, such as anti-mouse LC3 and anti-mouse inducible nitric oxide synthase (iNOS) Abs were purchased from Sigma (St. Louis, MO, USA) and AbCam (Cambridge, England), respectively.

### Culture of Bone Marrow-Derived Dendritic Cells and Macrophages

Bone marrow-derived DCs were cultured according to the protocols adapted from Lutz et al. (24). Briefly, bone marrow cells (BMC) were flushed aseptically from femurs and tibia of mice. For DC culture, cells were grown in RPMI-1640 (Invitrogen Life Technologies, Eugene, OR, USA) containing FBS (10%) (GIBCO, Grand Island, NY, USA) with penicillin (100 U/ml), streptomycin (100 mg/ml), and l-glutamine (100 mM) and supplemented with recombinant murine granulocyte-macrophage-colony-stimulating factor (rGMCSF) (2 ng/ml) and recombinant murine interleukin (rIL)-4 (4 ng/ml). Cultures were maintained in a humidified atmosphere at 5% CO_2_/37°C. The medium was replenished after 3 days.

### Stimulation of BMDCs

On 6 days, BMDCs were incubated with biotin conjugated anti-CD40 Ab (CD40A) or isotype control Abs (IgG2a,κ) (4 μg/10^6^ cells) for 30 min on ice followed by cross-linking with equivalent concentration of streptavidin, under similar conditions. The cells were washed and then plated in culture plates (5 × 10^5^ cells/ml) containing either medium alone or medium containing TLR-4 agonist (5 ng/ml). Suitable controls such as cells alone, cells stimulated with anti-CD40 Abs or isotype-matched Abs, with streptavidin or with TLR-4 agonist alone were also included in all experiments. For macrophages, BMC were grown in RPMI-1640 + FBS-10% and replenished with L929 supernatant (SN) (20%), as a source of macrophage-colony-stimulating factor (M-CSF). These cells were stimulated as described above for BMDCs.

### Immunofluorescent Staining

Dendritic cells harvested at 24 h were resuspended in FACS buffer (2% FBS, 2 mM sodium azide in PBS). To inhibit non-specific staining, cells were incubated with anti-CD16/32 Ab for 25 min at 4°C. Later, cells were stained with fluorochrome-conjugated Abs specific for mouse CD80, CD86, MHCII, or isotype-matched control Abs at a recommended concentration (0.5 μg/10^6^ cells). The cells were fixed with 1× paraformaldehyde. Regular steps of washing were followed at each step. Data were collected using FACS ARIA and analyzed with the BD DIVA software. The integrated MFI (IMFI) was calculated by multiplying the MFI with percentage of cells.

### Cytokines Estimation

Cytokines *viz* IL-6, IL-12, TNF-α, and IFN-γ were detected in culture SNs at indicated time points (24 h) by standard ELISA, according to manufacturer’s instruction.

### Culturing of Mycobacterium

*Mtb* strains (H37Rv, H37Ra) were provided by Dr. VM Katoch, National JALMA Institute for Leprosy and Other Mycobacterial Diseases, Agra and GFP-H37Ra by Dr. P Gupta, IMTECH, Chandigarh, India. *Mtb* was cultured in Middlebrook 7H9 broth containing glycerol (0.2%) and Tween-80 (0.05%), supplemented with albumin, dextrose, and catalase. The viability of bacteria was determined by plating on Middlebrook 7H11 medium supplemented with oleic acid, albumin, dextrose, and catalase and counting the number of colony-forming units (CFUs).

### *In Vitro* Infection with Mycobacterium

Dendritic cells and macrophages were infected with *Mycobacterium smegmatis* or *Mtb* at MOI 1:5 and harvested after 3 or 4 h, respectively. The extracellular bacteria were removed by four to five times washing with 1× PBS. *M. smegmatis* and *Mtb*-infected DCs were stimulated with C40.T4 [CD40A (4 μg/10^6^ cells) and TLR-4L (5 ng/ml)] and cultured in the presence of amikacin (2 μg/ml) for 16 or 24 h, respectively, in 48 W plate to kill extracellular bacteria. Where indicated, isoniazid (INH) (25 μg/ml) and rifampicin (RIF) (0.5 μg/ml) were added along with C40.T4.

### CFUs Determination

Bacterial growth in DCs and macrophages was quantified after 24 h of infection. Cell SNs were removed and 0.1% saponin was added to lyse the cells and plating was done with 10-fold serial dilution on 7H11 agar plate. The colonies were enumerated 3 weeks after incubation at 37°C/5% CO_2_.

### Nitric Oxide Production

Supernatants of *Mtb*-infected DCs were harvested after 48 h of infection and nitric oxide (NO) was measured by Griess method (25, 26). Briefly, SNs (50 μl) were incubated with an equal volume of Griess reagent for 5 min at RT. Later, absorbance was measured at 550 nm.

### Antigen Uptake

Dendritic cells were stimulated with C40.T4 for 24 h. The cells were harvested, washed, and then pulsed with HRP (50 μg/ml). Antigen was chased for 30–60 min and uptake was arrested by adding chilled PBS (1×) followed by transferring cells on ice. Cells were washed extensively with ice cold PBS-FBS-1%. Subsequently, cells were lysed using Tris-HCl (10 mM) and Triton X-100 (0.05%) for 30 min on ice, with intermittent vortexing. Intracellular HRP was estimated colorimetrically in the cell lysates using OPD-H_2_O_2_ chromogen-substrate. Concentration of HRP uptake was measured with respect to standard. Cells maintained at 4°C were used as control. HRP activity in test samples was suitably normalized with controls.

For confocal analysis, DCs were stimulated as mentioned above for 24 h. The cells were infected with GFP-*Mtb* (H37Ra) at (MOI 1:5) for 4 h, washed extensively (4×) with ice cold PBS (1×) and fixed with paraformaldehyde (4%). The cells were washed and placed on poly-l-lysine coated cover slips and imaged using Zeiss Confocal Laser microscope. *Z*-stacks were taken to exclude the interference of extracellular bacteria. Results were examined by image analysis software.

For bacterial uptake estimation through CFU, DCs were stimulated with C40.T4 for 24 h. Later, DCs were harvested and infection was given at MOI (1:5) for 4 h. It was followed by lysis of cells with 0.1% saponin and plating was done with 10-fold serial dilution on 7H11 agar plate. The colonies were enumerated 3 weeks after incubation at 37°C/5% CO_2_.

### CD40 and Beclin siRNA Knock Down Assay

Dendritic cells were incubated with ON TARGET plus CD40 and beclin siRNA (Dharmacon, Chicago, IL, USA), according to manufacturer’s protocol. Briefly, DCs were incubated with CD40 (20 nM) and beclin (20 nM) siRNA in serum-free media in the presence of transfection reagent 2 for 72 h. Later, level of inhibition in CD40 expression was estimated through flowcytometry and beclin at mRNA level by RT-qPCR. CD40 or beclin knock down DCs were infected with *Mtb* following the same protocol as mentioned above.

### Inhibition Assay with NO Inhibitor

*Mtb*-infected DCs were stimulated through C40.T4 in the presence or absence of iNOS inhibitor (*N*-monomethyl-l-arginine) (1 μM) for 24 h. Later, cells were lysed and CFU plating was done to quantify the viable bacteria.

### Expression of LC3 by Western Blotting

Dendritic cells were stimulated through C40.T4 for 2 h to detect LC3 and 18 h after *Mtb* infection for iNOS expression by Western blotting. Cells were washed and lysed with radioimmunoprecipitation assay (RIPA) buffer. Cellular extracts (40 μg protein) were subjected to SDS-polyacrylamide gel electrophoresis and transferred to a nitrocellulose membrane. Blots were incubated with anti-LC3 and iNOS Abs, followed by HRP-conjugated secondary Abs. Regular steps of incubation and washing were followed at each stage. Membranes were developed using a chemiluminescent reagent and subsequently exposed to film (Pharmacia-Amersham, Freiburg, Germany). Starving DCs were used as positive controls.

### Expression of LC3 by Immunofluorescence

Dendritic cells stimulated through C40.T4 were fixed on poly-l-lysine coated cover slips for 15 min. Later, cells were fixed with paraformaldehyde (4×) for 10 min followed by treatment with Tween-20 (0.1%) for 30 s. To block non-specific sites, cells were incubated with BSA (5%) for 3 h, followed by rabbit anti-mouse LC3 Ab for 4 h. Subsequently, cells were incubated with anti-rabbit FITC for 1 h. Regular washings were done followed at each step. The cells were imaged through confocal microscopy. Starving DCs were also kept as positive control. Punctas were counted manually.

### RT-qPCR for the Quantification of Beclin

Total RNA was isolated by Trizol reagent from DCs incubated with beclin siRNA according to the manufacturer’s instructions (Dharmacon, Chicago, IL, USA). RNA was quantified with the help of NanoDrop. A260/A280 ratio of all samples was in the range of 1.90–2.00. Intactness of RNA samples was determined with the help of formaldehyde denaturing agarose gel-electrophoresis. DNA contamination from RNA samples was removed by amplification grade DNase (Sigma, St Louis, MO, USA). Briefly, RNA samples (1 μg) were incubated with DNase (1U) for 15 min in the reaction buffer. After the incubation, DNase activity was terminated by stop solution (Sigma, St Louis, MO, USA). Furthermore, the samples were heated to 70°C for 10 min to inactivate DNase activity. Results are represented in the form of re-expression (fold) relative to untreated controls. Analysis was done by comparative Ct method, whereas Ct values were normalized against house-keeping control actin. RT-qPCR and data analysis were done by Realplex Mastercycler (Eppendorf, Hamburg, Germany).

### Therapeutic Strategy

Mice were aerosol challenged with *Mtb* for the deposition of 100 CFUs in lungs. At 24 h after aerosol challenge, three mice were sacrificed for quantification of pathogen delivery to lungs by measuring CFU in lung homogenates. Mice were found to be infected with the 100–120 CFU of H37Rv in their lungs. After 21 days, animals were administered twice with the CD40A (50 μg/300 μl/mouse) in conjunction with TLR-4L (1 μg/300 μl/mouse) (C40.T4) i.p. with an interval of 15 days (18). The control groups were injected with either CD40A (50 μg/300 μl/mouse) or TLR-4L (1 μg/300 μl/mouse) or placebo (PBS). After 50 days of aerosol challenged, animals were sacrificed and the bacterial burden was enumerated in the lungs by CFU counting.

### Isolation of Lymphocytes and Phenotypic Analysis

Mice were treated with C40.T4 or controls with CD40A or TLR4L or PBS after 21 days of aerosol challenged with *Mtb*. Fifty days later, mice were sacrificed. The animals were perfused with cold PBS-heparin and spleen and mediastinal lymph nodes were harvested and single cell suspension was prepared. Briefly, lymphocytes from spleen were prepared by lysing RBCs with ACK lysis buffer (NH_4_Cl 0.15 M, KHCO_3_ 10 mM, EDTA 88 mM), washed 3× with PBS and resuspended in RPMI-1640-FBS-10%. Viability of the cells was assessed by trypan blue dye-exclusion method. The experiments were performed to detect intracellular cytokines and effector/central memory markers on T cells upon *in vitro* restimulation with purified protein derivative (PPD).

### Adoptive Transfer of CD4 T Cells and CD8 T Cells into Sub-Lethally Irradiated Mice

Splenocytes of *Mtb*-challenged mice treated with C40.T4 were *in vitro* stimulated with PPDs to expand *Mtb* reactive T cells. Later, CD4 T cells and CD8 T cells were purified by negative selection using MACS sorter, as per manufacturer’s instructions (BD Biosciences, San Diego, CA, USA). CD4 T cells or CD8 T cells (5 × 10^6^) were administered into recipient mice after 24 h of sub-lethal irradiation (500R). Within 24 h of cell transfer, recipient mice were aerosol challenged with *Mtb* (27).

For *in vitro* assay, purified CD4 T cells and CD8 T cells isolated as mentioned above were co-cultured with *Mtb*-infected DCs for 48 h. Later, cells were lysed with 0.1% saponin and plating was done with 10-fold serial dilution on 7H11 agar plate. The colonies were enumerated 3 weeks after incubation at 37°C/5% CO_2_.

### Statistical Analysis

The GraphPad Prism software program was used to perform all statistical analysis. Statistical testing was performed by one way ANOVA for group analysis and Student’s *t*-test for comparing two groups. Differences were considered significant at a level of *p* < 0.05.

## Results

### Signaling through C40.T4 Induced the Maturation and Activation of DCs

Although, signaling to DCs was delivered through CD40, CD80, and CD86 molecules but CD40-stimulated DCs exhibited optimal activation (Figure S1A in Supplementary Material). Furthermore, CD40-mediated signaling was significantly amplified in conjunction with TLR-4 (Figure S1B in Supplementary Material). Hence, in the subsequent experiments, CD40 and TLR-4 (C40.T4) were used to activate DCs. We used Abs to CD40 (CD40A) and highly pure LPS (EK ultrapure) as an agonist for TLR-4 (TLR-4L) signaling. The optimum concentration of CD40A (4 μg/10^6^ cells) and TLR-4L (5 ng/ml) was selected on the basis of dose-dependent response in releasing IL-6 and IL-12 (Figure S1B in Supplementary Material). IL-6 and IL-12 are the major cytokines produced by DCs in response to infection by intracellular pathogens. These cytokines have potent effect on T cells activation and other cells of immune system. Therefore, they are considered as an important bridge between innate and adaptive immunity. Upregulation of costimulatory molecules is a characteristic feature of mature DCs (28). Interestingly, DCs triggered through C40.T4 showed significant enhancement in IMFI of CD80 (*p* < 0.05), CD86 (*p* < 0.05) and MHCII (*p* < 0.05) molecules (Figures S1C–E in Supplementary Material). No change was observed with isotype-matched control Abs of CD40. It was of concern for us whether the influence of signaling through C40.T4 is restricted to DCs alone or can stimulate the macrophages as well. Similarly, we observed that triggering C40.T4 substantially (*p* < 0.01) enhanced the production of IL-6 by macrophages (Figure S2A in Supplementary Material).

**Figure 1:**
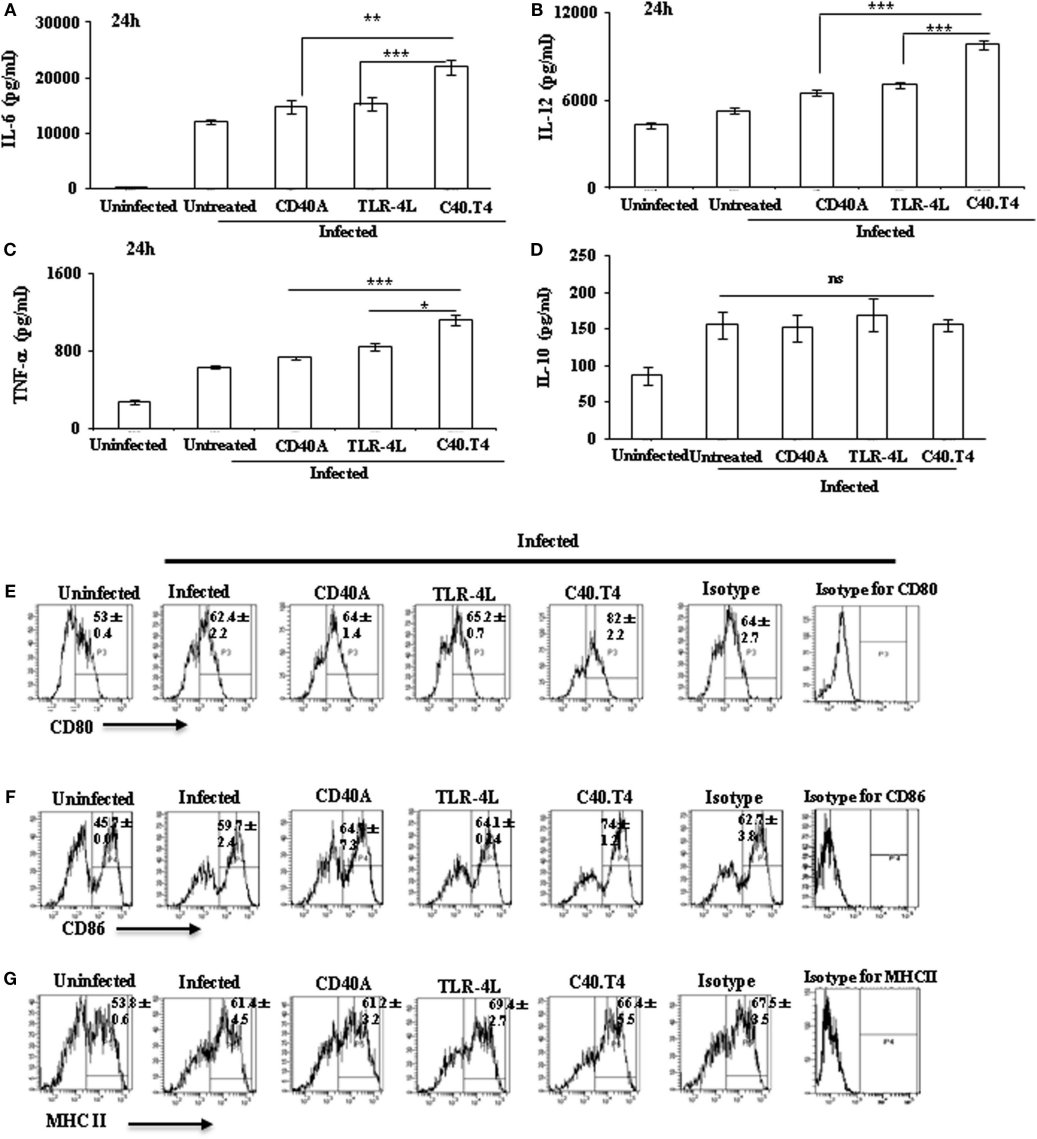
**Signaling through C40.T4 enhanced the production of cytokines and expression of CD80, CD86, and MHCII on *Mtb*-infected DCs**. *Mtb*-infected DCs were stimulated through C40.T4 for 24 h. Control cultures were also set using CD40A, TLR4L, isotype-matched controls, and medium alone. Later, SNs were harvested for the detection of **(A)** IL-6; **(B)** IL-12; **(C)** TNF-α; **(D)** IL-10 by ELISA and expressed as pg/ml. Data shown as mean ± SD are normalized with their respective isotype-matched controls. CD11c^+^ DCs were phenotyped by flowcytometry for the expression of **(E)** CD80 (*p* < 0.001); **(F)** CD86 (*p* < 0.01); **(G)** MHCII. Data expressed as mean ± SD of percent positive cells are representative of three independent experiments. “*,” “**,” and “***” indicate *p* < 0.05, *p* < 0.01, and *p* < 0.001, respectively.

The aim of the current study was to activate *Mtb*-infected DCs through C40.T4 and, consequently, to control the growth of the pathogen. We observed that C40.T4 stimulation improved the potency of infected DCs to produce cytokines, such as IL-6 (*p* < 0.001), IL-12 (*p* < 0.0001), and TNF-α (*p* < 0.001) (Figures 1A–C). No change was noted in IL-10 (Figure 1D). Similarly, infected macrophages stimulated with C40.T4 augmented the secretion of IL-6 (*p* < 0.05) (Figure S2B in Supplementary Material). Furthermore, *Mtb*-infected DCs stimulated through C40.T4 expressed considerably higher levels of costimulatory molecules CD80 (*p* < 0.01) and CD86 (*p* < 0.01), as compared to control (Figures 1E–G). However, not much change was observed in the expression of MHCII. It may be due to saturation in the expression of MHCII on DCs upon infection with *Mtb*.

**Figure 2:**
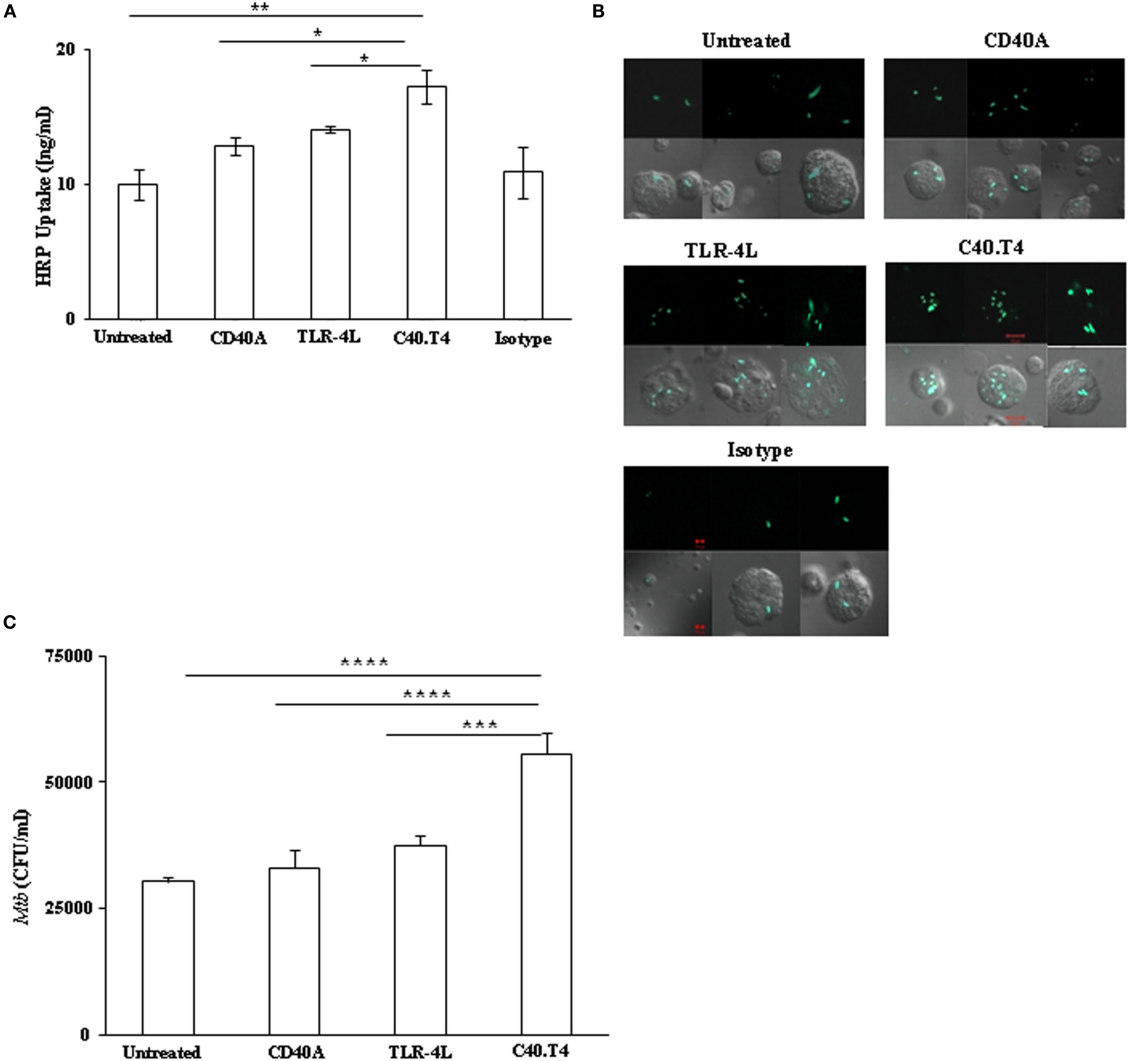
**C40.T4 augmented the antigen uptake capacity of DCs. (A)** C40.T4 stimulated DCs were pulsed with antigen HRP for 60 min. Cells were washed and lysed to measure HRP uptake by colorimetry. Values were normalized with experimental blank and control cells kept on ice. **(B)** C40.T4 activated DCs were co-cultured with GFP-expressing *Mtb* (*gMtb*) for additional 4 h and were imaged through confocal microscopy. Each image represents the cells from three different fields. **(C)** C40.T4 activated DCs were infected with *Mtb* for 4 h. Later, mycobacterial uptake was enumerated by CFUs plating. Results are expressed as mean ± SD **(A,C)**. Data are the representative of three individual experiments. “*” and “**” indicate *p* < 0.05 and *p* < 0.01, respectively.

Dendritic cells are highly efficient in antigen uptake (28). Therefore, we next studied whether the C40.T4 stimulation enhanced the capacity of DCs for antigen uptake. Interestingly, C40.T4 signaling showed significant (*p* < 0.05) augmentation in the antigen (HRP) uptake as observed by colorimetric method (Figure 2A). It is known that DCs endocytose HRP chiefly through fluid phase pinocytosis driven by constitutive membrane ruffling activity and traces of it is taken up by receptor-mediated endocytosis (29, 30). It suggests that DCs activated through C40.T4 stimulation exhibited substantial enhancement in HRP uptake through pinocytosis. Furthermore, we studied the phagocytic ability of DCs by confocal microscopy and CFU plating assay. Our results demonstrate higher uptake of GFP^+^*Mtb* by DCs upon stimulation through C40.T4 and noteworthy (*p* < 0.05) increase in the *Mtb* uptake by confocal microscopy and CFU plating, respectively (Figures 2B,C). These results suggest that the signaling of DCs through C40.T4 improves their endocytic capacity.

### C40.T4 Triggered DCs Efficiently Constrained the Intracellular Survival of *Mtb*

**Figure 3:**
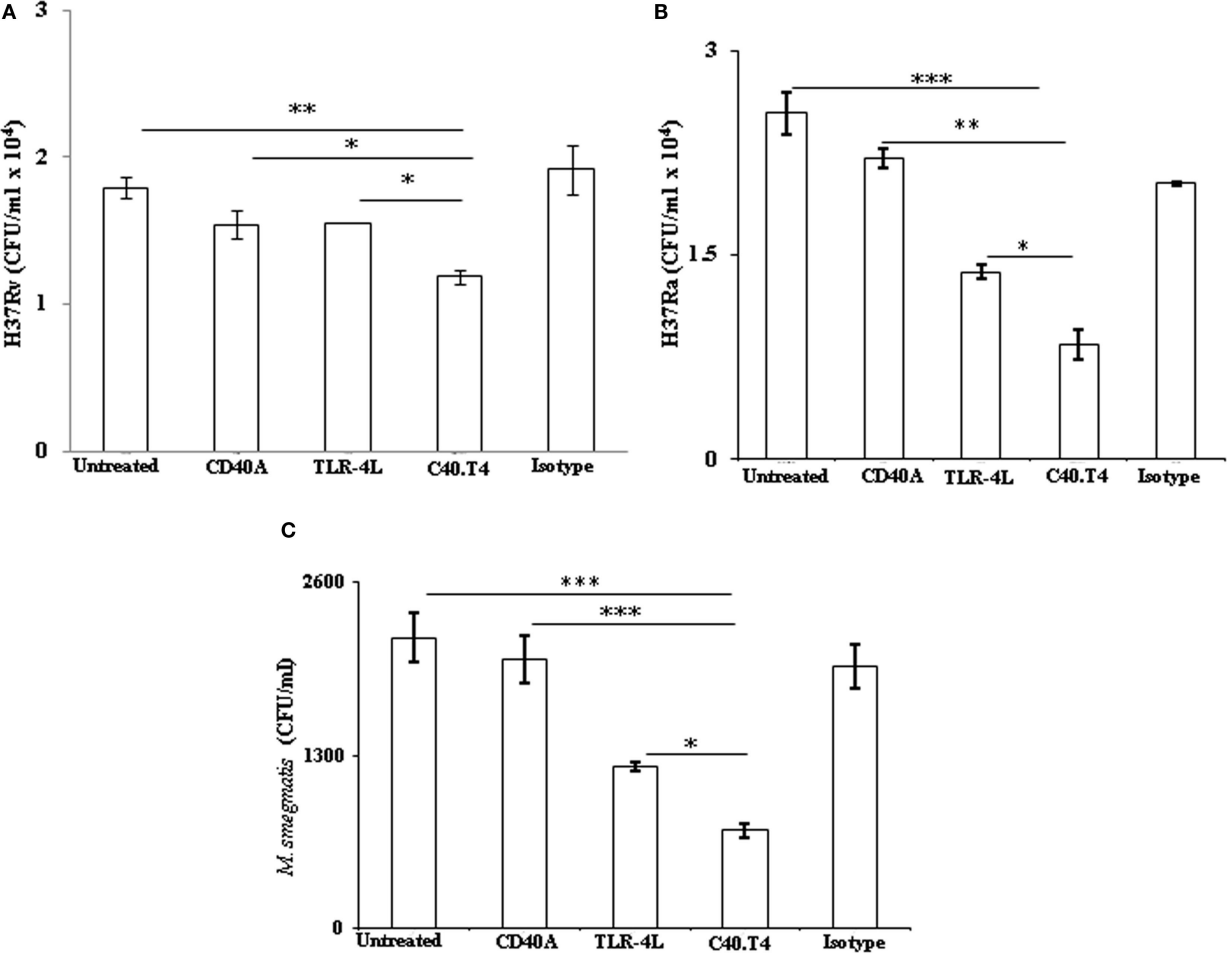
**Signaling delivered through C40.T4 potentiate DCs ability to restrict *Mtb* growth**. DCs were infected with mycobacterial strains H37Ra, H37Rv, and *M. smegmatis* followed by stimulation through C40.T4 for 24 h. Later, mycobacterial survival was enumerated by CFUs plating for **(A)** H37Rv on 21 days; **(B)** H37Ra on 21 days; **(C)** *M. smegmatis* on 3 days. Data are represented as the mean ± SD of three independent experiments. “*” indicate *p* < 0.05.

Next, we were interested to study the effect of C40.T4 on the bactericidal activity of DCs. *Mtb*-infected DCs were triggered through C40.T4 for 24 h. We observed enhanced bactericidal activity of C40.T4 stimulated DCs, as evidenced by significant killing of virulent strain (H37Rv) of *Mtb* (*p* < 0.05). These results were further substantiated using non-virulent strains of mycobacteria, such as H37Ra (*p* < 0.05) and *M. smegmatis* (*p* < 0.05) (Figures 3A–C). Not much change was observed in CD40 or TLR-4 or isotype-matched controls. Similar results were observed in the case of macrophages (Figure S2C in Supplementary Material). It is important to mention that the difference in uptake of *Mtb* as shown in (Figure 2C) had no impact on their bactericidal activity. In the case of phagocytic assay, DCs were first stimulated with C40.T4 and then infected with *Mtb*. By contrast, bactericidal activity of DCs was demonstrated by first infecting them with *Mtb* and then stimulating through C40.T4.

### DCs Deficient in CD40 and TLR-4 Fails to Restrict the Growth of *Mtb*

**Figure 4:**
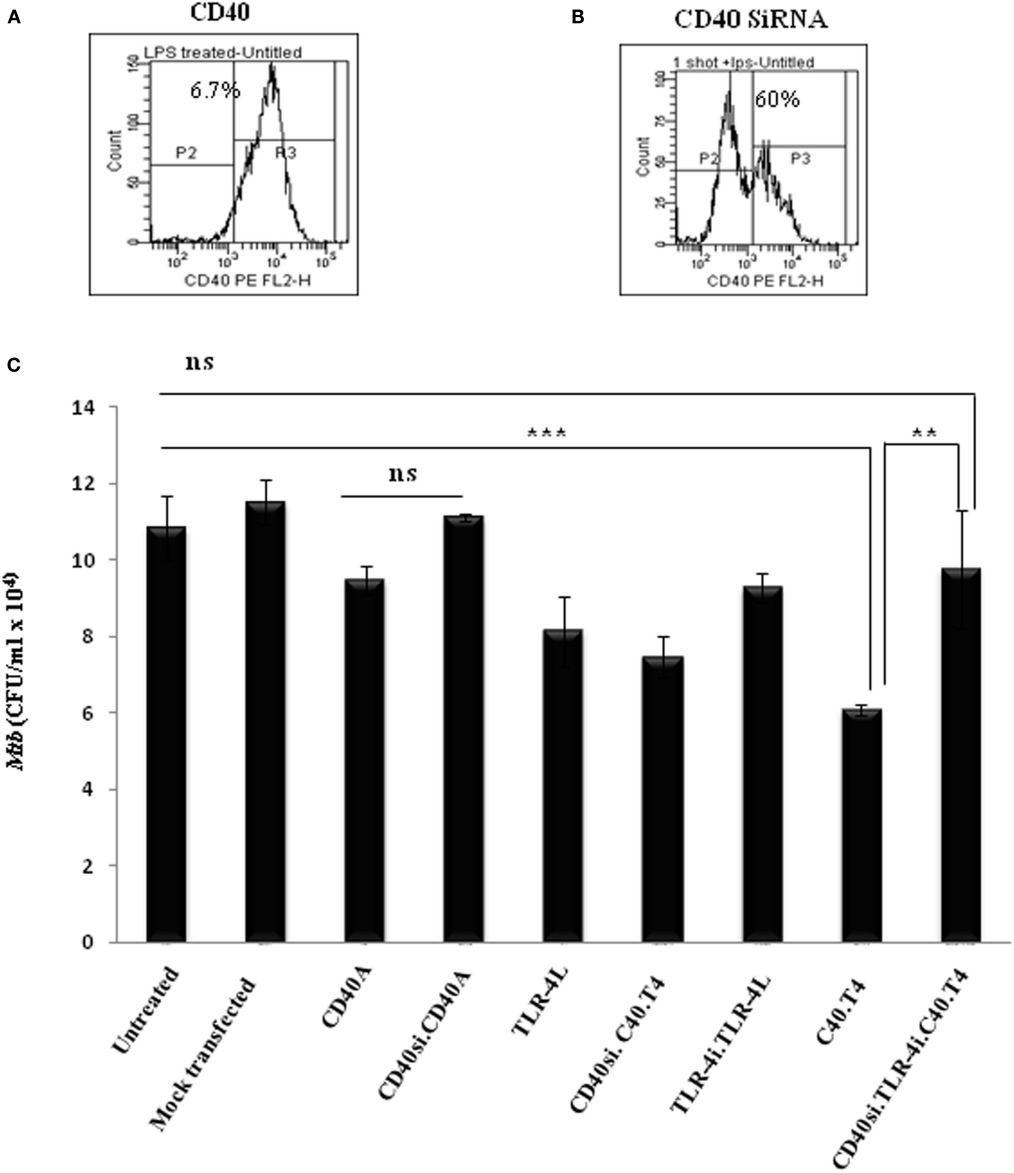
**Knockdown of CD40 in the presence of TLR-4 inhibitor (TLR-4^i^) establishes the specificity of signaling delivered through C40.T4 in inhibiting the survival of *Mtb***. DCs were treated with CD40 siRNA (CD40si) for 48 h. Expression of CD40 on DCs was monitored by flowcytometry. Number in histograms indicates the percentage of CD40 negative population of DCs **(A)** wild-type cells; **(B)** CD40 knock down cells. **(C)** CD40 knock down and wild-type DCs were infected with *Mtb* followed by stimulation through C40.T4 in presence or absence of TLR-4^i^ for 24 h. Later, cells were lysed and bacterial burden was enumerated on 21 days by CFU counting. Data shown are mean ± SD and representative of two independent experiments. CD40si: CD40 knockdown by SiRNA; TLR-4i: TLR-4 inhibitor.

It was quite important for us to check the specificity of signals delivered through CD40 and TLR-4. CD40 gene in DCs was knock down by siRNA (CD40^KD^). Efficacy of the assay, as observed by flowcytometry, was 60% (Figures 4A,B). In addition, the specificity of TLR-4 signaling was established using TLR-4 inhibitor (TLR-4^i^) CLI-095. Next, CD40^KD^ DCs infected with *Mtb* were stimulated with CD40A and TLR-4L in the presence or absence of TLR-4^i^. Interestingly, such CD40^KD^ DCs cultured in the presence of TLR-4^i^ followed by their stimulation through C40.T4 showed no decline in the growth of *Mtb*, when compared with *Mtb*-infected DCs triggered through C40.T4. We also observed no change in the control DCs with or without mock transfection (Figure 4C). Hence, establishing the specificity of these experiments.

### Adjunct Therapy with C40.T4 and Anti-TB Drugs Substantially Enhances Bactericidal Potency of DCs

**Figure 5:**
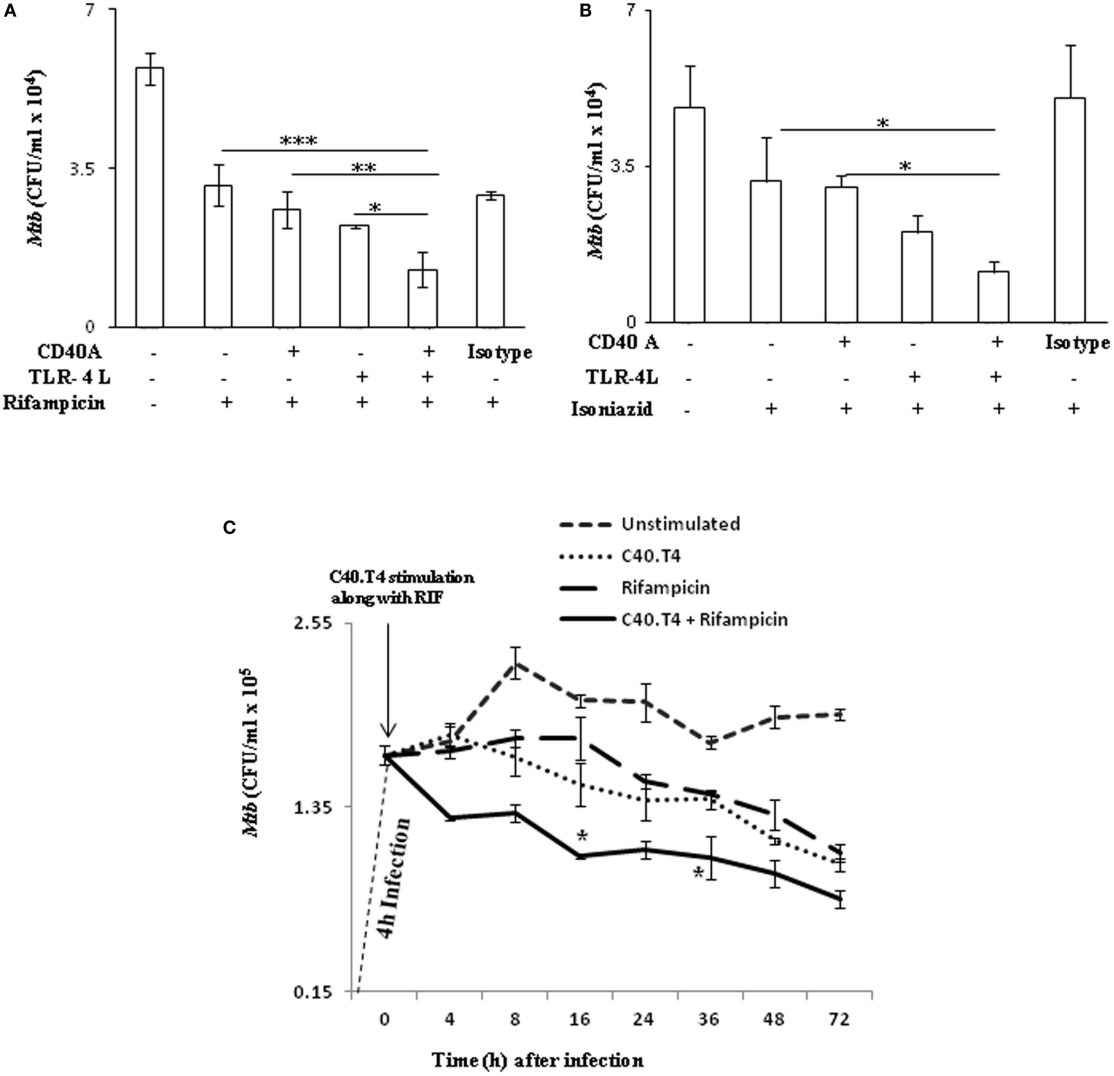
**C40.T4 triggering enhances the potency of RIF and INH to kill intracellular *Mtb***. DCs infected with *Mtb* were stimulated through the indicated combinations of C40.T4, CD40, TLR-4 with **(A)** RIF (24 h) **(B)** INH (24 h); **(C)** RIF (0–72 h). Later, cells were lysed and CFUs were monitored on 21 days. Data represented as mean ± SD **(A–C)** are of three independent experiments. “*,” “**,” and “***” indicate *p* < 0.05, *p* < 0.01, and *p* < 0.001, respectively.

The lengthy drug-regime for treating TB patients not only inflicts side-effects but also other serious problems like emergence of drug-resistant strains of *Mtb*. Therefore, we hypothesized to explore adjunct therapy with drugs in combination with immunomodulators to minimize the dose and duration of anti-TB drugs and increase their bactericidal activity. *Mtb*-infected DCs activated through C40.T4 considerably bolstered the bactericidal potency of anti-TB drugs RIF (*p* < 0.001) and isoniazid (*p* < 0.05), in comparison to drugs alone (Figures 5A,B). It may be concluded from these results that adjunct therapy of boosting immunity *via* CD40 and TLR-4 may be an important strategy to enhance the killing efficacy of drugs.

Importantly, our time kinetics study established that adjunct therapy employing immunomodulators and drugs could substantially reduce the duration of drug regime for killing *Mtb* (Figure 5C). *Mtb*-infected DCs activated through C40.T4, upon treatment with RIF exhibited reduction in the intracellular burden of *Mtb* (1.8 × 10^5^ to 1 × 10^5^) at a significantly lesser time (16 h) than treatment with RIF alone (72 h) (Figure 5C). Thus, our observation establishes the importance of adjunct therapy utilizing CD40A and TLR-4L along with drugs. This adjunct therapy could be further explored as immunotherapeutic regimen for reducing the duration of TB treatment.

### C40.T4 Induced Bactericidal Mechanism Is Autophagy Dependent

**Figure 6:**
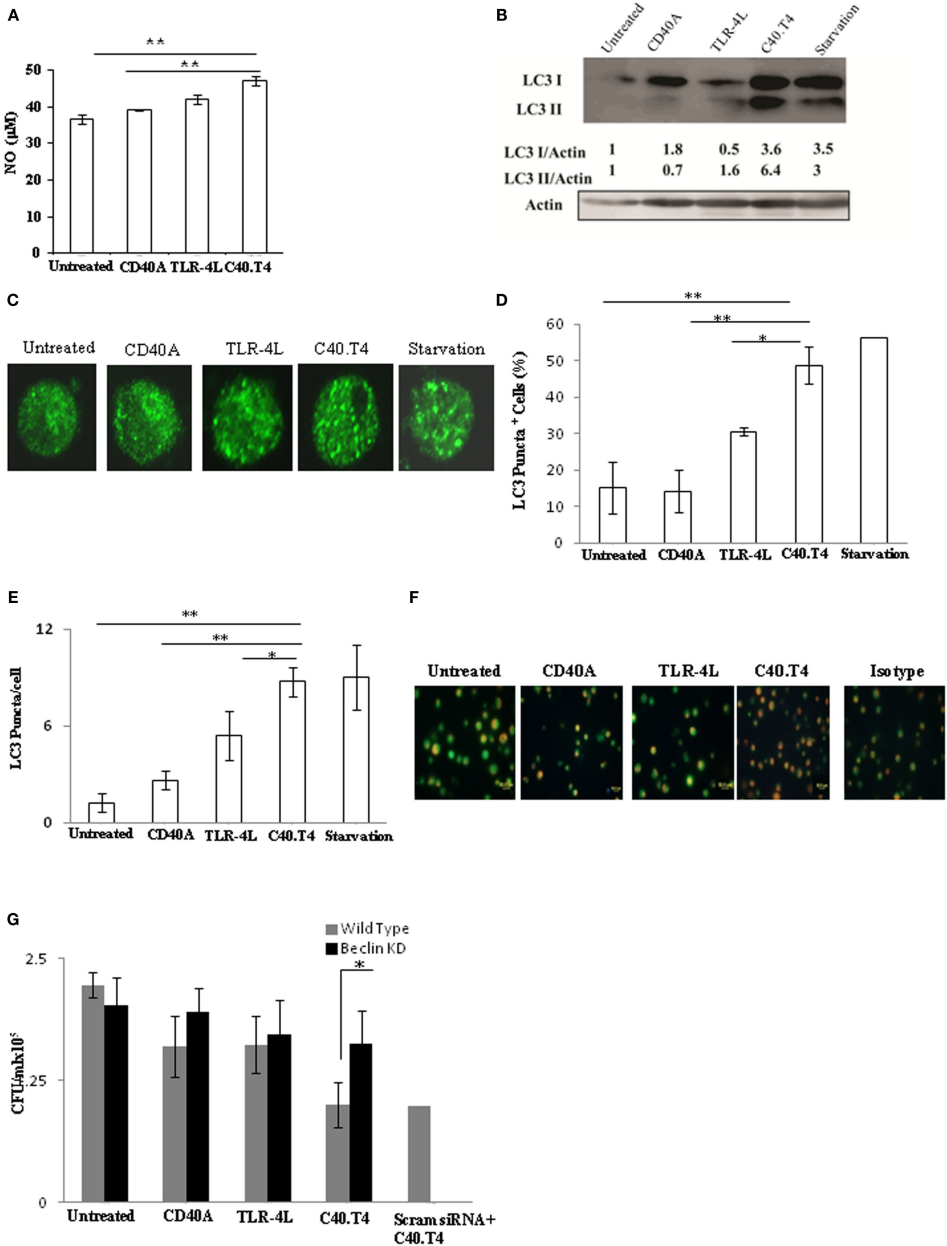
**Triggering through C40.T4 showed augmentation in autophagy**. DCs infected with *Mtb* were stimulated through C40.T4, CD40, TLR-4 for 24 h and assessed for **(A)** NO in SNs by Greiss assay. **(B)** Expression of LC3 was detected in the whole cells lysates of DCs stimulated for 2 h through C40.T4 by immunoblotting. Actin was used as a loading control. Densitometric data show the LC3I/actin and LC3II/actin ratio. **(C)** LC3 puncta formation was demonstrated by immunofluorescence staining. Starved DCs were used as a positive control; **(D,E)** Bar graphs depict the percentage of LC3 puncta positive cells and LC3 puncta per cells, respectively. **(F)** DCs were stimulated through C40.T4 for 5 h were later incubated with acridine orange for 15 min to visualize autophagosomes by fluorescence microscopy (40×). Orange dots indicate the acidic vacuoles. **(G)** Beclin^KD^ DCs were infected with *Mtb* followed by stimulation with C40.T4. Later, cells were lysed and bacterial burden was quantified by CFU assay. Data represented as the mean ± SD **(A,D,E,G)**; immunoblotting **(B)**; fluorescent microscopy **(C,F)**; are of 2–3 independent experiments. “*” and “**” indicate *p* < 0.05 and *p* < 0.01, respectively.

Involvement of NO is a classical mechanism related with the killing of intracellular pathogens. Hence, we thought to monitor the change in the NO secretion. Importantly, magnitude of NO secretion was significantly (*p* < 0.01) enhanced in *Mtb*-infected DCs triggered through C40.T4, as compared to untreated or CD40 or TLR-4 triggered DCs (Figure 6A). Furthermore, our Western blotting experiments also showed considerable enhancement in the expression of iNOS (Figure S3A in Supplementary Material). We confirmed the specificity of NO on the survival of *Mtb* by culturing C40.T4 activated DCs with iNOS inhibitor (*N*-monomethyl l-arginine). We observed the recovery of viable bacteria in the presence of iNOS inhibitor (Figure S3B in Supplementary Material). However, only marginal restoration in the survival of *Mtb* was achieved, indicating the involvement of an additional mechanism. Hence, we thought to monitor autophagy, which is considered to be quite crucial in inhibiting the intracellular survival of *Mtb* (31, 32). Intriguingly, C40.T4 triggered DCs exhibited significantly higher conversion of LC3I to LC3II, a hallmark phenomenon of autophagy (Figure 6B). This information was corroborated with intracellular LC3 staining for puncta formation (Figures 6C–E). Furthermore, acidic vacuoles were stained with acridine orange dye to show the induction of autophagy (Figure 6F). In addition, we have knocked down the expression of beclin in DCs through siRNA prior to *Mtb* infection (Figure S3C in Supplementary Material). We observed that beclin knock down DCs if triggered through C40.T4 fails to restrict the *Mtb* growth (Figure 6G). These results suggest that C40.T4-induced autophagy significantly contributes in constraining the *Mtb* growth.

### Immunization with C40.T4 Bolstered the Immune Response and Reduced *Mtb* Burden in Mice

**Figure 7:**
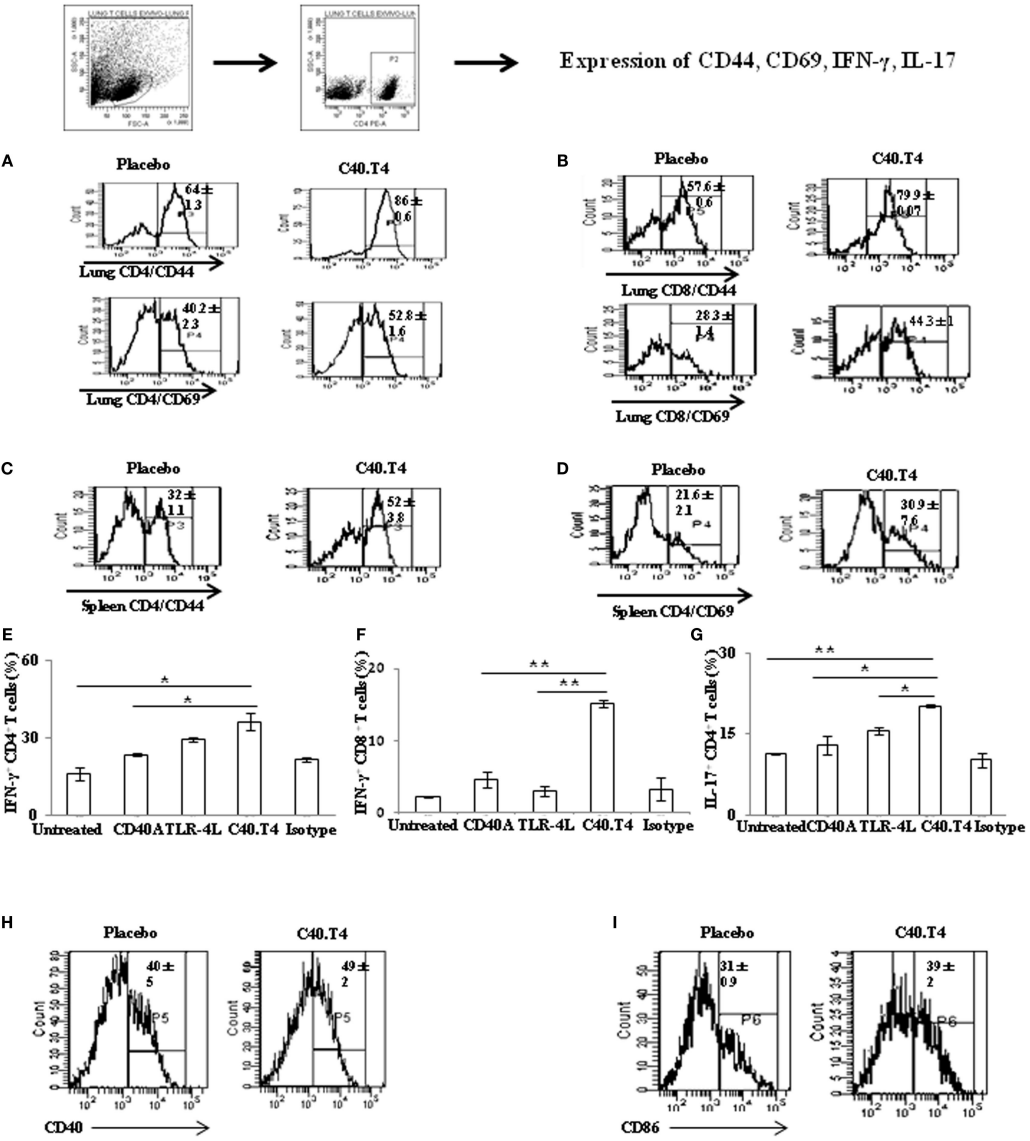
**C40.T4 treatment bolstered the T cells immunity in mice exposed to *Mtb***. *Mtb*-infected animals were administered C40.T4. After 50 days, mice were sacrificed and lymphocytes were isolated and cultured with purified protein derivative (6 μg/ml) for 48 h. Later, cells were stained for the expression of **(A)** CD44/CD69 on CD4 T cells isolated from the lungs; **(B)** CD44/CD69 on CD8 T cells isolated from the lungs; **(C,D)** CD44/CD69 on CD4 T cells isolated from the spleens. Number in the histograms indicates the percentage of CD44^hi^ and CD69^hi^ on CD4 and CD8 gated population. Splenocytes stimulated *in vitro* with PPD were stained for expression of **(E)** IFN-γ on CD4 T cells; **(F)** IFN-γ on CD8 T cells; **(G)** IL-17 on CD4 T cells. The bar diagrams illustrate percent population and results expressed as mean ± SD. DCs isolated from the lungs of *Mtb*-infected animals treated with C40.T4 were stained for the expression of **(H)** CD40; **(I)** CD86 and on CD11c + DCs. Number in the histogram indicates the mean ± SD of CD11c^+^ DCs expressing CD40 and CD86 population. The data are representative of two independent experiments (*n* = 4 mice/group). “*,” “**,” and “***” indicate *p* < 0.05, *p* < 0.01, and *p* < 0.001, respectively.

Finally, we used this strategy in the experimental model of TB. The animals were aerosol challenged with *Mtb* and 21 days later inoculated with CD40A and TLR-4L. After 50 days, mice were sacrificed. The cells were isolated from the lungs and spleen and cultured with PPD for 48 h. Later, we monitored the expression of activation markers *viz*. CD44 and CD69 on CD4 T cells. Interestingly, we observed that animals injected with C40.T4, displayed higher levels of CD44 and CD69 on CD4 T cells (Figures 7A–D). Similar results were noted in the case of CD8 T cells (Figure 7B). Control groups constituting of individually administered CD40A or TLR-4L showed marginal change in the expression of activation markers. It implies the importance of synergism between C40.T4 molecules in reinvigorating immunity during *Mtb* infection.

CD4 T cells and CD8 T cells are crucial in providing protection against *Mtb* (33). IFN-γ released by CD4 and CD8 T cells play a pivotal role in restricting the growth of *Mtb* (34). IFN-γ^–/–^ mice are fatally susceptible and humans deficient for IFN-γ or IFN-γR are prone to *Mtb* infection (35). Although, IFN-γ is necessary for immunity against TB, but its presence alone is not sufficient for protection. Recently, IL-17 has been proven to be efficient in reducing the bacterial burden (36, 37). Therefore, we examined the expression of IFN-γ and IL-17. Mice inoculated with anti-CD40 Abs and TLR-4L showed predominance of Th1 and Th17 response on *in vitro* stimulation with PPD, as evident by significantly higher production of IFN-γ (*p* < 0.05) and IL-17 (*p* < 0.05), respectively (Figures 7E–G). Similar results were noted for IFN-γ (*p* < 0.01) release by CD8 T cells (Figure 7F). Furthermore, we observed significant upregulation in the expression of CD86 (*p* < 0.05) and CD40 on DCs isolated from the lungs of *Mtb*-infected animals injected with C40.T4 (Figures 7H,I).

**Figure 8:**
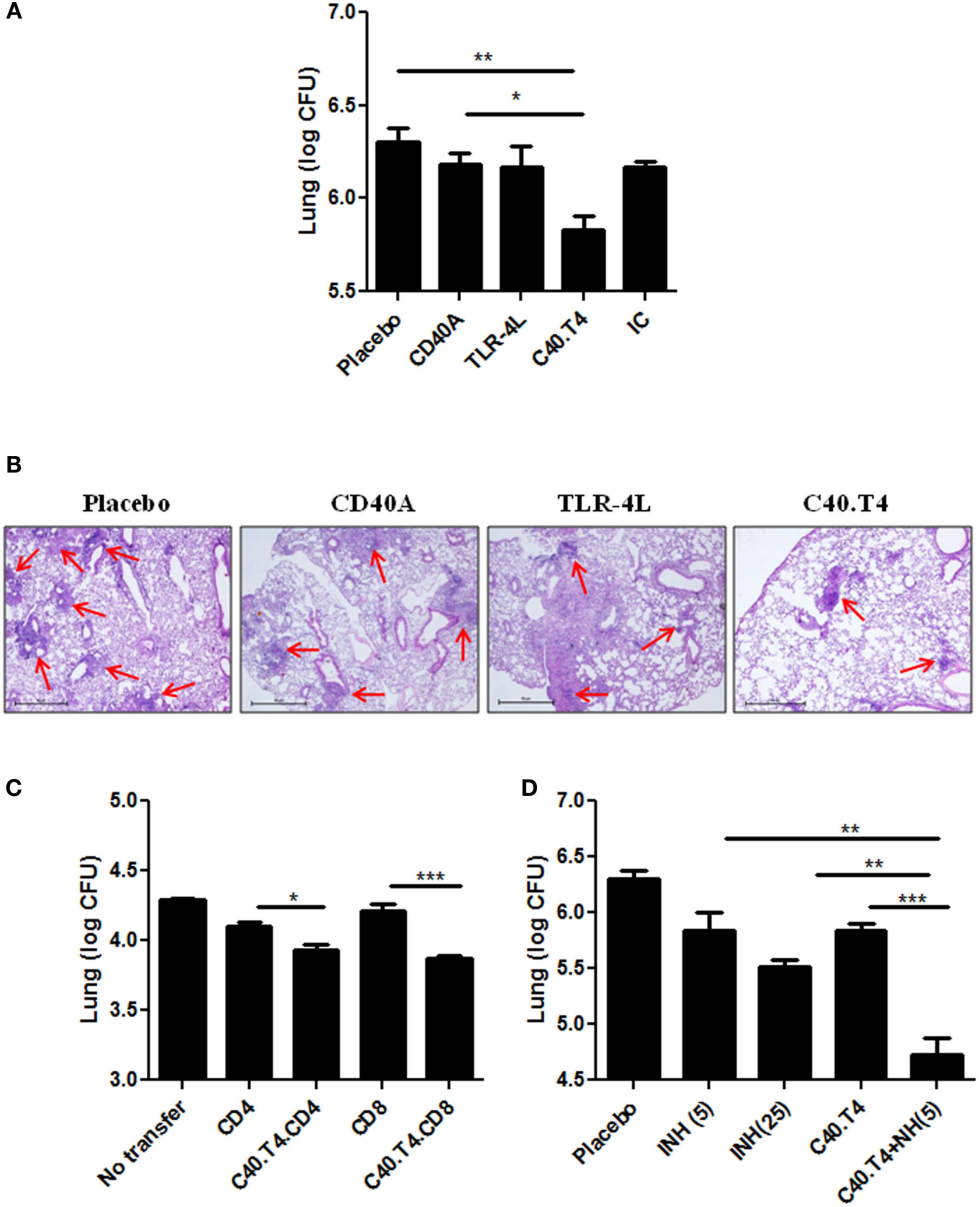
**Immunomodulation through C40.T4 reduces the bacterial burden in the lungs of *Mtb*-challenged mice and enhances the potency of isoniazid. (A)** Mice infected with *Mtb* were immunized with C40.T4. Later, animals were sacrificed and mycobacterial load in the lungs was enumerated by CFUs plating; **(B)** Photomicrographs (40×) of H & E stained lung sections. **(C)** CD4 and CD8 T cells were purified from C40.T4 treated and *Mtb*-challenged mice. Later, CD4 or CD8 T cells were adoptively transferred into sub-lethally irradiated mice. After 12 h, mice were aerosol challenged with *Mtb*. The bacterial burden in the lungs was enumerated on 15 days by CFUs plating. Labeling of “*x*” axis signifies CD4 or CD8: adoptive transfer of either CD4 or CD8 T cells isolated from *Mtb*-challenged mice; C40.T4.CD4 or C40.T4.CD8: adoptive transfer of either CD4 or CD8 T cells isolated from *Mtb*-challenged and C40.T4-treated mice. **(D)** Animals infected with *Mtb* were administered with C40.T4 and two doses of INH (INH5: 5 mg/kg bwt, INH25: 25 mg/kg bwt of mouse). Later, mice were sacrificed and mycobacterial load was enumerated in the lungs by CFUs plating. Data represented as mean ± SEM are from two independent experiments with (*n* = 4mice/group). Control groups of mice were administered PBS, CD40A, TLR-4L. “IC”: isotype control. “*,” “**,” and “***” indicate *p* < 0.05, *p* < 0.01, and *p* < 0.001, respectively.

Next, we monitored the CFU burden in the lungs of C40.T4 treated mice. We observed significant (*p* < 0.01) decline in the mycobacterial burden compared to control mice treated with PBS, TLR-4L and CD40A (Figure 8A). Furthermore, the data were validated by histopathology samples. We observed that C40.T4 treated lungs showed less granulomatous lesions. The lungs were less consolidated with more normal alveolar structures. The number of granulomas were decreased in the lungs of C40.T4 immunized mice, as compared to placebo control (Figure 8B). These experiments showed the potential of C40.T4 immunotherapy in boosting the immunity of the *Mtb*-infected animals along with reduction in the bacterial load.

### Adoptive Transfer of CD4 and CD8 T Cells Showed Significant Reduction in *Mtb* Burden

Next, we have demonstrated the *vis-à-vis* role of CD4 T cells and CD8 T cells in limiting *Mtb* growth. Hence, we purified CD4 T cells and CD8 T cells from *Mtb* aerosol challenged mice treated with C40.T4 and adoptively transferred in sub-lethally γ-irradiated animals. Mice were aerosol challenged with *Mtb*. After 15 days, animals were sacrificed and significant reduction (*p* < 0.05) in the bacterial load in the lungs of mice was noticed. Similar results were observed with CD8 T cells (*p* < 0.001) (Figure 8C). Likewise, to prove the influence more precisely, CD4 T cells and CD8 T cells were co-cultured with *Mtb*-infected DCs. We observed significant decline in the CFUs in cultures incubated with CD4 T cells (*p* < 0.05) and CD8 T cells (*p* < 0.05) (Figures S4A,B in Supplementary Material). These results suggest that C40.T4 triggering augments the T cell response, which contributes in inhibiting the growth of bacterium.

### C40.T4 Adjunct Therapy with INH Significantly Potentiates *In Vivo* Efficacy of INH in Restraining the Growth of *Mtb*

*Mtb*-infected mice treated with INH in conjunction with C40.T4 showed significant (*p* < 0.001) reduction in the mycobacterial load in the lungs (Figure 8D). Most importantly, the dose of 5 mg/kg bwt of INH administered to mice as an adjunct therapy with C40.T4 imparted considerably (*p* < 0.05) better decline in the CFUs, as compared to 25 mg/kg bwt of INH alone. It is worth to mention here that the C40.T4 + INH was administered only twice over a period of 30 days after *Mtb* challenge. These results support our hypothesis that C40.T4 significantly reduces the dose of anti-TB drug INH.

## Discussion

Dendritic cells are the bridging component of both innate and adaptive immunity and their function is regulated by their differentiation, maturation, and activation status (38). However, in-spite of their sentinel role in eliciting immune response, DCs role is limited due to their suboptimal maturation *in vivo* in certain diseases, such as cancer and AIDS (39). Furthermore, pathogens, such as *Mtb*, leishmania, cancer, etc., hamper their function and, therefore, paralyze the immune system (11). Therefore, to boost the immune response, it is quite crucial to rescue DCs from pathogens or tumors that interfere in their performance.

CD40 in conjunction with TLR-4 has been reported in substantially augmenting DCs activity to regress tumor growth and optimally activate T cells (7, 10, 19). Thus, indicating the immunotherapeutic potential of C40.T4 in bolstering the function of DCs and thereby systemic immunity. To the best of our knowledge, the role of C40.T4 has not been monitored in any infectious disease. Henceforth, we investigated the combinatorial role of C40.T4 in the activation of DCs, thus hampering the growth of *Mtb*. We also studied the immunotherapeutic potential of C40.T4 as an adjunct therapy with anti-TB drugs INH and RIF to reduce the dose and duration of the TB drug regimen in influencing the survival of *Mtb*. Following major findings have emerged from this study when signaling of DCs was delivered through C40.T4 (i) upregulation of the activation markers CD80, CD86, MHCII; (ii) robust production of IL-6, IL-12, TNF-α; (iii) augmented ability of DCs to phagocytose antigen; (iv) enhanced lung immunity and reduced *Mtb* burden; (v) significant decrease in the dose and duration of the drugs and increment in their bactericidal potency.

Our *in vitro* data demonstrate the role of signaling delivered through C40.T4 in the activation of *Mtb*-infected DCs. DCs are inferior in bactericidal activity and, therefore, boosting their killing efficacy will strengthen their function. The phenotypic markers of C40.T4 triggered DCs (TNF-α^hi^, IL-12^hi^, IL-6^hi^) indicated that they acquired enough potential to kill *Mtb*. *Mtb* infects both DCs and macrophages (40). Similar to DCs, infected macrophages also restrict the growth of *Mtb* by activating bactericidal mechanism, such as NO production. In addition, macrophages are the major contributor in forming the structured granulomas, responsible in controlling the *Mtb* growth (41). Therefore, it is important to bolster the function of macrophages to control *Mtb* growth. Noteworthy, C40.T4 signaling also augmented the bactericidal activity of macrophages, as evidenced by restriction in the intracellular survival of *Mtb* (Figure S2C in Supplementary Material), but need further investigation to elucidate the mechanism adapted by CD40 and TLR-4 macrophages to kill *Mtb*.

To establish infection, *Mtb* evades the immune mechanism. Blocking of phago-lysosome fusion is one of the strategies utilized by *Mtb* to hampers the antigen presentation by DCs and macrophages (42). Henceforth, it is important to design strategy that can overcome the inhibition in phagolysosme fusion. Autophagy is a phenomenon that targets the cytoplasm content and damaged organelle to autophagosome, which in turn fuse with lysosome. In addition, autophagy and NO are considered to be major pathways involved in the clearance of array of pathogens, including *Mtb* (31, 43). Interestingly, CD40 and TLR-4 signaling showed augmentation in autophagy and marginal increase in NO release.

Th1 cells confer protective immunity against *Mtb* through production of effector cytokine IFN-γ. In addition, Th17 cells protect against *Mtb* (44). Besides IFN-γ, current vaccination strategies for TB also show the importance of IL-17 cytokine (45). More interesting findings came out with the observation that IL-17 is crucial to induce protection in hyper-virulent but not lesser-virulent strains of *Mtb* (36). Noteworthy, administration of C40.T4 in *Mtb*-challenged animals augments Th1 and Th17 response. It suggest that increase in the production of both IFN-γ and IL-17 with C40.T4 administration may play a vital role in generating protective immunity against clinical isolates of *Mtb*.

Though, our *in vitro* data support that C40.T4 induces the activation of DCs and macrophages but improved host immunity in infected animals could be an outcome of involvement of many other cells. Since, B cells also express CD40 and recently B cells have been shown to regulate the level of granulomatous reaction, cytokine production, and T cells response (46). It needs further investigation that how other cells respond to CD40 and TLR-4 signaling *in vivo* and may contribute in mediating protection against *Mtb*.

In addition, we monitored the role of C40.T4 as an adjunct therapy with anti-TB drugs in curbing the growth of *Mtb*. Although, current TB drug regime is extremely efficient but the major impediment is long-duration of at least 6-month treatment. Therefore, inevitably giving rise to several side-effects and providing ample time to mycobacterium in developing drug resistance (47). Nevertheless, remarkable potency of TB drugs still cannot be overlooked. A number of studies have been attempted to enhance the efficacy of TB drugs for better outcome and were considered beneficial (48). Based on published reports with potential role of CD40 and TLR-4 in other disease models, we explored them in reinforcing the host immunity against *Mtb* (7). Furthermore, the added advantage of C40.T4 immunotherapy was that it significantly decreased the dose of anti-TB drugs. Animals delivered fivefold lesser dose (5 mg/kg/bwt) of INH bimonthly showed significant reduction in the bacterial burden in the lungs, against the recommended daily treatment of 25 mg/kg/bwt (49). There is a fundamental advantage of INH-C40.T4 adjunct therapy since the drug would kill the replicating *Mtb* and C40.T4 would take care of drug induced immunosuppression and also adjuvantage host immunity to kill non-replicating quiescent microorganism. Furthermore, INH-C40.T4 strategy will be beneficial in (i) subsiding the drug inflicted side-effects because of low dosage; (ii) modulating host immunity; (iii) restraining the emergence of drug-resistance; and (iv) reducing the dose and duration of the drug regime. The idea of implementing this approach of drug as an adjunct therapy with immunomodulators to kill *Mtb* and concurrently bolstering host-immunity may pave a way for designing immunotherapeutic strategy to effectively control TB.

## Author Contributions

Concept or design of the work (JA and NK); or the experiments (NK, SP, AV, and MA); analysis, or interpretation of data for the work (NK and JA).

## Conflict of Interest Statement

The authors declare that the research was conducted in the absence of any commercial or financial relationships that could be construed as a potential conflict of interest.
