# Supplementary material for: Stimulation through CD40 and TLR-4 Is an Effective Host Directed Therapy against Mycobacterium tuberculosis
Source: Front Immunol. 2016 Sep 27;7:386. doi: 10.3389/fimmu.2016.00386 (PMC5037235; doi:10.3389/fimmu.2016.00386)
Supplement: Supplementary file 1 [file presentation_1.ppt]

## Slide 1
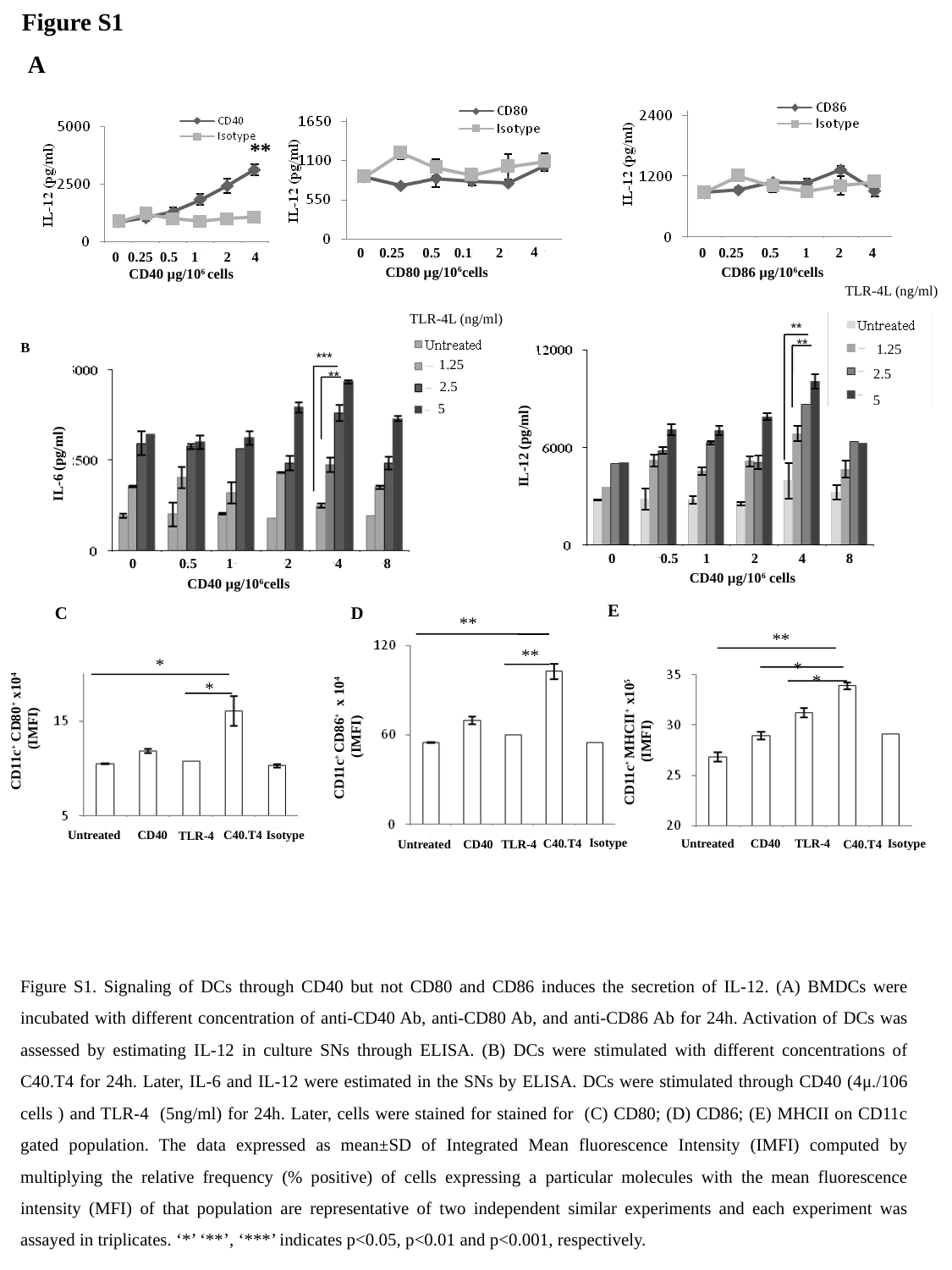

Figure S1
A
0
0.25
0.5
1
2
4
4
0
0.25
0.5
0.1
2
CD80 µg/106cells
0
0.25
0.5
1
2
4
CD40 µg/106 cells
**
CD86 µg/106cells
TLR-4L (ng/ml)
IL-12 (pg/ml)
0
0.5
1
2
4
8
CD40 µg/106 cells
TLR-4L (ng/ml)
IL-6 (pg/ml)
0
0.5
1
2
4
8
CD40 µg/106cells
**
**
B
 1.25
***
1.25
 2.5
**
2.5
 5
5
E
C
D
**
**
CD11c+ CD86+ x 104
 (IMFI)
Isotype
C40.T4
CD40
Untreated
TLR-4
**
*
*
CD11c+ MHCII+ x105
 (IMFI)
CD40
Isotype
Untreated
TLR-4
C40.T4
*
*
CD11c+ CD80+ x104
(IMFI)
CD40
Untreated
C40.T4
Isotype
TLR-4
Figure S1. Signaling of DCs through CD40 but not CD80 and CD86 induces the secretion of IL-12. (A) BMDCs were incubated with different concentration of anti-CD40 Ab, anti-CD80 Ab, and anti-CD86 Ab for 24h. Activation of DCs was assessed by estimating IL-12 in culture SNs through ELISA. (B) DCs were stimulated with different concentrations of C40.T4 for 24h. Later, IL-6 and IL-12 were estimated in the SNs by ELISA. DCs were stimulated through CD40 (4μ./106 cells ) and TLR-4 (5ng/ml) for 24h. Later, cells were stained for stained for (C) CD80; (D) CD86; (E) MHCII on CD11c gated population. The data expressed as mean±SD of Integrated Mean fluorescence Intensity (IMFI) computed by multiplying the relative frequency (% positive) of cells expressing a particular molecules with the mean fluorescence intensity (MFI) of that population are representative of two independent similar experiments and each experiment was assayed in triplicates. ‘*’ ‘**’, ‘***’ indicates p<0.05, p<0.01 and p<0.001, respectively.

## Slide 2
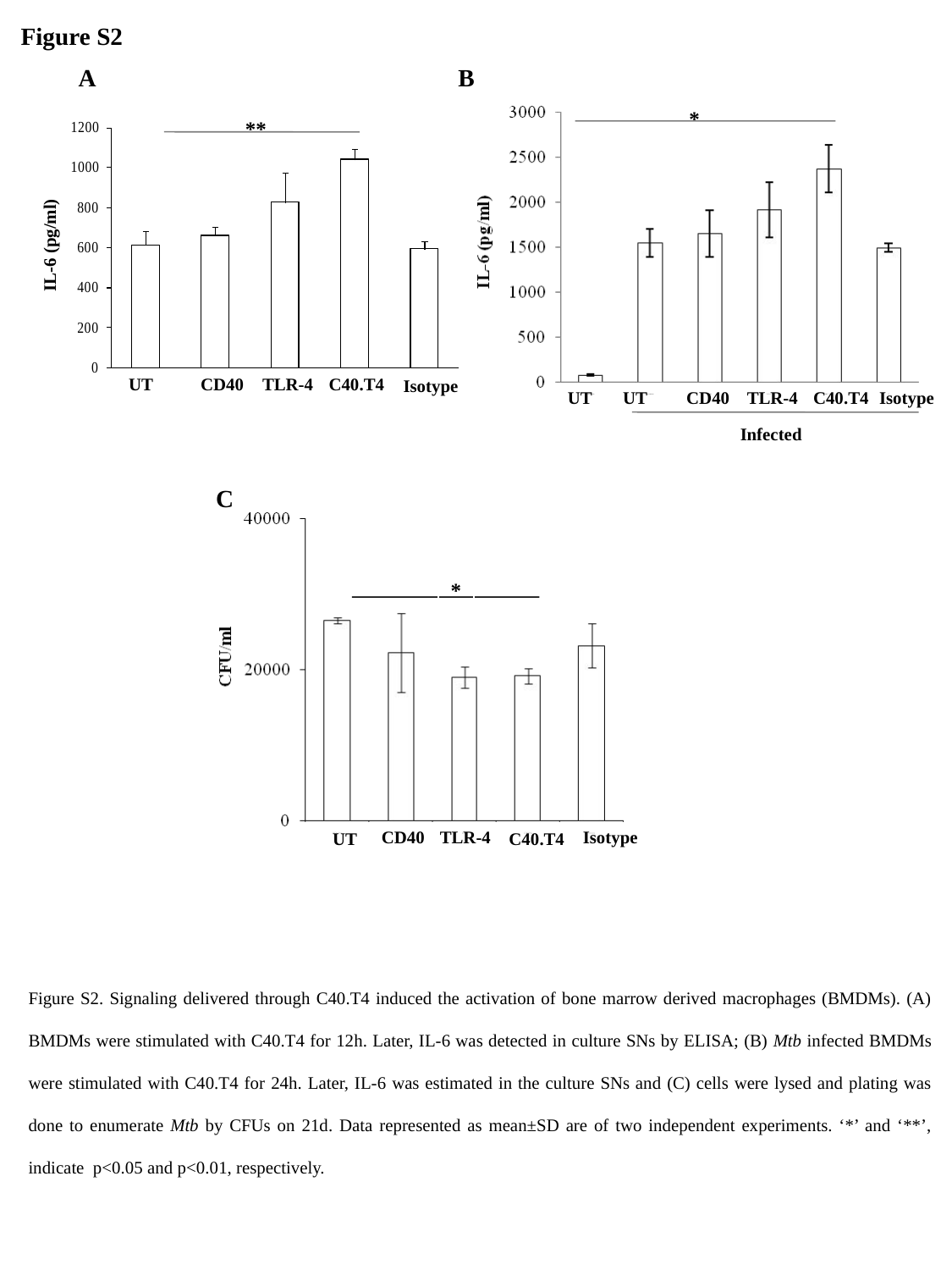

Figure S2
A
B
*
**
IL-6 (pg/ml)
UT
CD40
TLR-4
C40.T4
Isotype
UT
UT
CD40
TLR-4
C40.T4
Isotype
Infected
C
*
 CD40
TLR-4
 Isotype
UT
 C40.T4
Figure S2. Signaling delivered through C40.T4 induced the activation of bone marrow derived macrophages (BMDMs). (A) BMDMs were stimulated with C40.T4 for 12h. Later, IL-6 was detected in culture SNs by ELISA; (B) Mtb infected BMDMs were stimulated with C40.T4 for 24h. Later, IL-6 was estimated in the culture SNs and (C) cells were lysed and plating was done to enumerate Mtb by CFUs on 21d. Data represented as mean±SD are of two independent experiments. ‘*’ and ‘**’, indicate p<0.05 and p<0.01, respectively.

## Slide 3
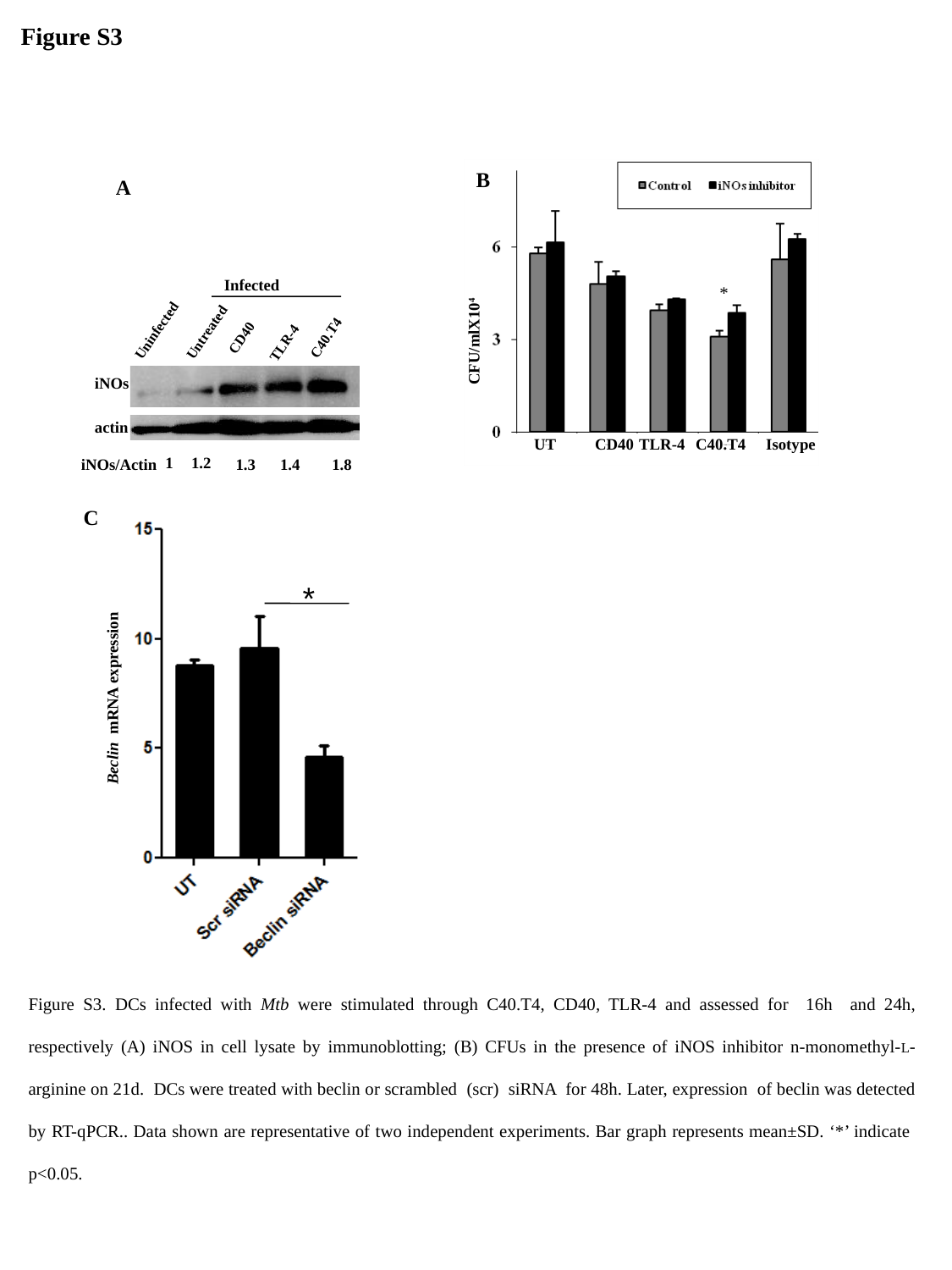

Figure S3
B
A
Untreated
C40.T4
Uninfected
CD40
TLR-4
iNOs
actin
1
1.2
1.4
iNOs/Actin
1.8
1.3
Infected
*
CFU/mlX104
UT
CD40
TLR-4
C40.T4
Isotype
C
*
Beclin mRNA expression
Figure S3. DCs infected with Mtb were stimulated through C40.T4, CD40, TLR-4 and assessed for 16h and 24h, respectively (A) iNOS in cell lysate by immunoblotting; (B) CFUs in the presence of iNOS inhibitor n-monomethyl-L-arginine on 21d. DCs were treated with beclin or scrambled (scr) siRNA for 48h. Later, expression of beclin was detected by RT-qPCR.. Data shown are representative of two independent experiments. Bar graph represents mean±SD. ‘*’ indicate p<0.05.

## Slide 4
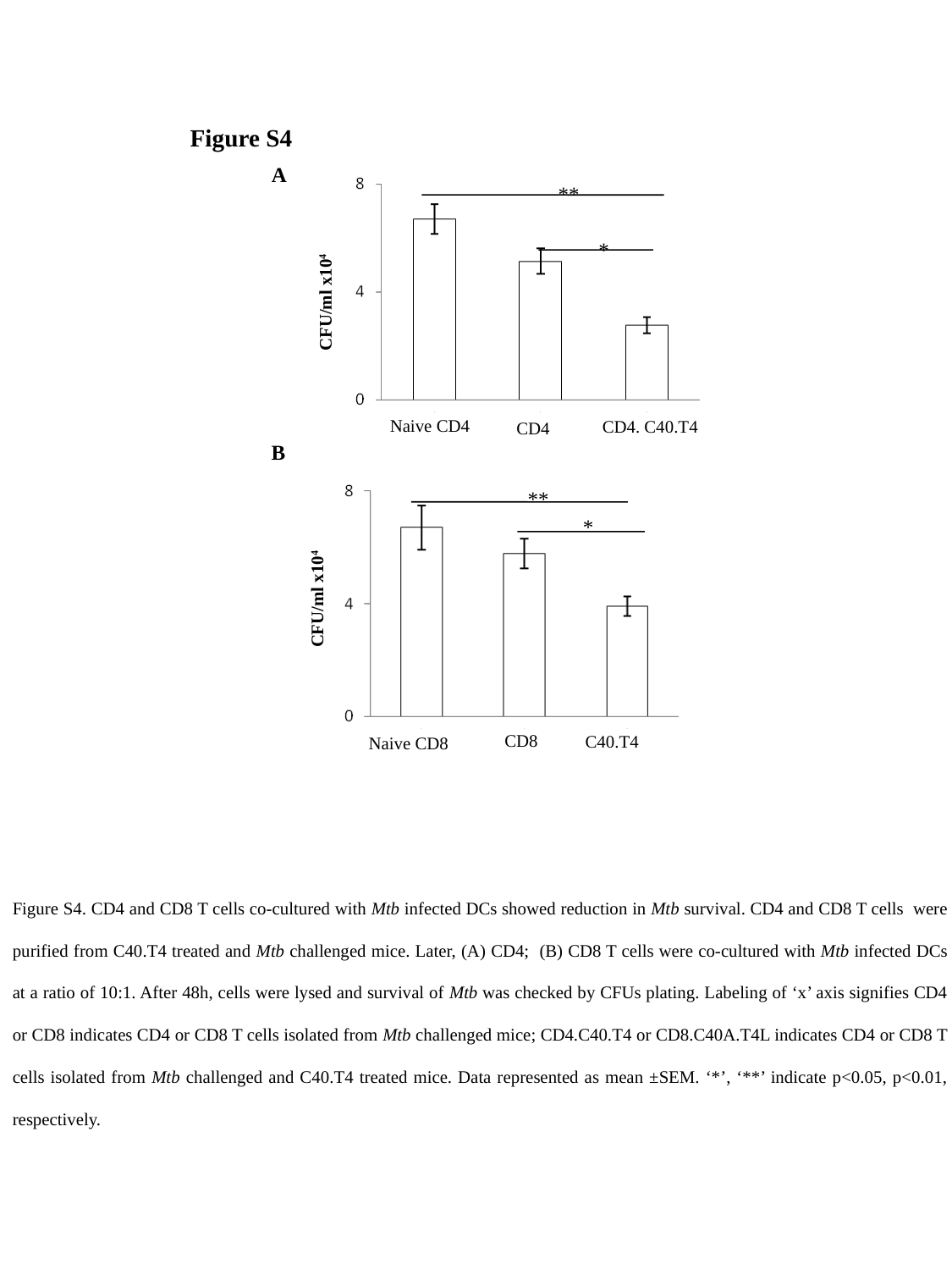

Figure S4
A
**
*
CFU/ml x104
Naive CD4
CD4. C40.T4
CD4
B
**
*
CFU/ml x104
CD8
C40.T4
Naive CD8
Figure S4. CD4 and CD8 T cells co-cultured with Mtb infected DCs showed reduction in Mtb survival. CD4 and CD8 T cells were purified from C40.T4 treated and Mtb challenged mice. Later, (A) CD4; (B) CD8 T cells were co-cultured with Mtb infected DCs at a ratio of 10:1. After 48h, cells were lysed and survival of Mtb was checked by CFUs plating. Labeling of ‘x’ axis signifies CD4 or CD8 indicates CD4 or CD8 T cells isolated from Mtb challenged mice; CD4.C40.T4 or CD8.C40A.T4L indicates CD4 or CD8 T cells isolated from Mtb challenged and C40.T4 treated mice. Data represented as mean ±SEM. ‘*’, ‘**’ indicate p<0.05, p<0.01, respectively.
